# Evaluation of trabeculated mass in patient with non compaction: do we need criteria reappraisal?

**DOI:** 10.1186/1532-429X-18-S1-P274

**Published:** 2016-01-27

**Authors:** Julien Frandon, Stéphanie Bricq, Monique Bernard, Alain Lalande, Alexis Jacquier

**Affiliations:** 1 grid.410529.b0000000107924829radiology, Grenoble University Hospital, France, Grenoble France; 2 grid.5613.10000000122989313Le2i, UMR CNRS 6306, Bourgogne University, Dijon, France; 3 Aix-Marseille University, CNRS, CRMBM UMR 7339, Marseille, France; 4 La timone, University Hospital, Marseille, France

## Background

There is no gold standard for the diagnosis of patients with Left ventricular non compaction (LVNC). There are 2D criteria in sonography with Jenni's criteria (*Jenni et al, Heart 2001*) and MRI with Petersen's criteria (*Petersen et al, Journal of the American College of Cardiology 2005*). Jacquier (*Jacquier et al, European Heart Journal 2010*) has proposed a 3 D evaluation of the non compacted mass with a threshold of 20% for the non compacted (NC)/compacted (C) mass ratio. They manually delineate the trabeculation and do not suppress blood. The aim of this study was to assess the effect of blood suppression with a semi-automatic software on this threshold.

## Methods

Segmentation of compacted (C) and non-compacted (NC) mass was performed on short axis SSFP CMR from 60 healthy participants and 15 patients with LVNC from the study by Jacquier et al, with a semi-automatic software. Papillary muscles were segmented using semi-automatic thresholding and included in the compacted mass. Blood was removed from trabeculae using the same threshold tool. The 15 patients with LVNC were also manually segmented for the calculation of NC mass. Inter-observer reproducibility was assessed by using Bland-Altman analysis (BA) and by computing the correlation coefficient in LVNC patients segmented with our software. Differences between manual and software segmentation were assessed using a Mann whitney U test. We compared the area under the 20% receiver operating characteristics (ROC) curve for NC/C mass ratio. We used ROC curves to determine the optimal cut off values for NC mass. Results were considered significant with a *p* < 0.05.

## Results

*Healthy subjects****:***

NC mass was 10.57 ± 3.89 g, C mass was 113.07 ± 27.27 g, NC/C was 9.51 ± 3.12 %.

*LVNC patients segmented manually:*

NC mass was 52.43 ± 24.47 g, C mass was 149,3 +/- 46,37 g, NC/C was 37.58 ± 15.68 %.

Area under ROC curve NC/C=20%: 0.98

Area under ROC curve NC=15g: 0.99

*LVNC patients segmented with our software including blood suppression:*

NC mass was 35.46 ± 17.02 g (p=0.03), C mass was 113.07 ± 27.27 g, NC/C was 24.97 ± 10.41 % (p=0.74). Volume of suppressed blood: 16.97 ± 9.56 mL. BA NC 0.96, BA C 0.98, BA NC/C 0.97.

Area under ROC curve NC/C=20%: 0.92 (p=0.03)

Area under ROC curve NC=15g: 0.98 (p=0.12)

## Conclusions

The method that we propose is highly reproducible and allows to calculate the exact mass of trabeculation. It could help for the diagnosis of LVNC with high area under curve but seems to be more discriminating with NC mass than with NC/C mass ratio.

